# Sociodemographic Factors and Health Insurance Coverage Are Associated with Invasive Breast Cancer in Tennessee: Appalachian and Non-Appalachian County Comparison

**DOI:** 10.1089/whr.2021.0136

**Published:** 2022-05-20

**Authors:** Faustine Williams, Lohuwa Mamudu, Charlotte J. Talham, Francisco A. Montiel Ishino, Martin Whiteside

**Affiliations:** ^1^ Division of Intramural Research, National Institute on Minority Health and Health Disparities, National Institutes of Health, Bethesda, Maryland, USA.; ^2^ Department of Public Health, California State University, Fullerton, California, USA.; ^3^ Tennessee Cancer Registry, Tennessee Department of Health, Nashville, Tennessee, USA.

**Keywords:** breast cancer, health disparities, sociodemographic factors, Appalachian Tennessee, non-Appalachian Tennessee

## Abstract

**Background:**

Tennessean women experience the 12th highest breast cancer (BC) mortality in the United States. Yet, few studies have examined BC outcomes among Tennessean women in and outside of Appalachia. We examined whether sociodemographic factors and health insurance status were associated with invasive BC in Tennessee by Appalachian and non-Appalachian county designation.

**Materials and Methods:**

Using the Tennessee Cancer Registry, we identified 52,187 women, aged ≥18, diagnosed with BC between 2005 and 2015. Multivariable logistic regression was performed to examine associations between invasive BC and sociodemographic characteristics, health insurance coverage, and county designation (Appalachian/non-Appalachian). Regression analyses stratified by county designation were subsequently performed.

**Results:**

In Tennessee, younger women had lower odds of invasive BC diagnosis (<45: odds ratio [OR] = 0.74, 95% confidence interval [CI] = 0.67–0.81; 55–64: OR = 0.91, 95% CI = 0.84–0.97) compared to women ≥65. Married Tennessean women had 12% (95% CI = 1.04–1.21) higher odds of invasive BC than single women. Further, both public (OR = 1.81, 95% CI = 1.41–2.33) and private (OR = 1.36, 95% CI = 1.06–1.76) health insurance were found to increase odds of invasive BC compared to no insurance/self-pay. Results from the subpopulation analyses were largely consistent with overall findings. In Appalachian counties, women on public health insurance had increased odds (OR = 1.42, 95% CI = 1.00–2.03) of invasive BC compared to uninsured/self-pay women, while in non-Appalachian counties, women insured both publicly (OR = 2.25, 95% CI = 1.57–3.24) and privately (OR = 1.68, 95% CI = 1.16–2.24) had increased odds of invasive BC.

**Conclusions:**

The results identify risk factors for Tennessean women in Appalachian and non-Appalachian counties whose malignancies evaded early detection, increasing risk of mortality.

## Introduction

The Appalachian Region of the United States spans 206,000 square miles and includes 432 contiguous counties across 13 states from New York to Mississippi.^1^ As of 2019, the region was home to roughly 25.7 million residents or 8% of the total U.S. population.^1^

One in four counties in Appalachia is considered rural according to the U.S. Department of Agriculture's Urban Influence Codes.^2^ The mountainous geography of Appalachia isolates many communities from health care resources and larger cities where healthy lifestyle and disease prevention messaging is more prevalent.^3^ The supply of primary care physicians per 100,000 residents in Appalachia is 12% lower than the national average and as much as 33% lower in some sub-regions.^2^ Additionally, poverty rates are higher and average household income and educational attainment lower in the Appalachian region than in the rest of the country.^4^ These circumstances leave many Appalachian counties economically distressed and medically underserved.

In 1992, the National Cancer Institute recognized stark health inequities in Appalachia by dedicating one of its 25 Community Network Programs to reducing cancer disparities in the region.^5^ In addition to geographic and socioeconomic obstacles to care, Appalachian residents experience higher prevalence of health and behavioral risk factors that may contribute to the observed disparities in the region. These include higher levels of obesity^6^ and smoking^7,8^ and less frequent screening recommendations by health care providers^9^ and use of screening services overall.^10^

Although cancer mortality rates in the United States have continuously decreased since the early 1990s, Appalachia bears a disproportionate burden of cancer incidence and mortality.^2,11–13^ For instance, between 2001 and 2011, breast cancer (BC) incidence in the non-Appalachian region saw a greater overall decrease than in the Appalachian region.^14^ Further, Appalachian counties experienced a smaller decline in BC mortality (17.5%) than non-Appalachian counties in the region (30.5%) between 1969 and 2007.^11,15^

BC remains the most diagnosed non-skin cancer among U.S. women, accounting for one in three diagnoses. It is also the second leading cause of cancer death among women, after lung cancer.^16^ Mammography is a preventive procedure used to detect BC at an early stage. Early detection is associated with easier and more successful treatment and higher rates of survival.^16^ The 10-year survival rate for patients diagnosed with ductal carcinoma *in situ* is around 98%^17,18^ whereas the rate falls to 84% for invasive BC diagnoses.^16^ Stark disparities persist in survival rates, however. Between 2011 and 2017, the 5-year survival rate for White women with invasive BC was 92% compared to 82% among Black women.^16^

**FIG. 1.**
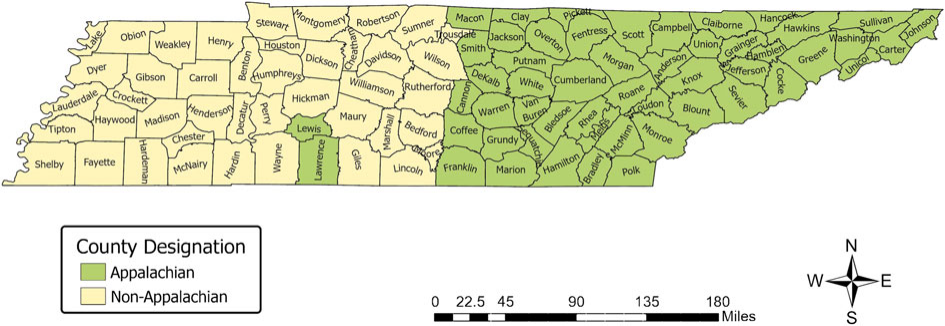
Appalachian and non-Appalachian county designations in Tennessee. *Note:* This map was created by the Health Disparities and Geospatial Transdisciplinary Research Lab at the National Institute on Minority Health and Health Disparities.

Tennessee is one of the largest and most diverse states in the Appalachian region in terms of race/ethnicity, income, and rurality. Fifty-two of Tennessee's 95 counties are classified as Appalachian (Fig. 1). The eastern portion of the state falls within Appalachia while the western portion is largely non-Appalachian, and the demographics of the juxtaposed regions differ widely.

Around 43% of Tennessee's 6.8 million residents live in Appalachian Tennessee. The median age of residents of Appalachian Tennessee is 42 compared to 37 in the non-Appalachian Tennessee region.^4^ Both Appalachian (86.9%) and non-Appalachian (63.4%) Tennessee are predominantly non-Hispanic White, although, non-Hispanic Black individuals comprise only 5.5% of the Appalachian Tennessee population compared to 25.2% of non-Appalachian Tennessee.^4^ Additionally, the proportion of residents without health insurance in Tennessee is 9.5% and 9.8% in Appalachia and non-Appalachia, respectively.^4^

BC disparities are particularly prevalent in Tennessee. The BC incidence rate per 100,000 individuals in Tennessee (123.1) is below the national average (126.9), however, the death rate is higher (21.8 compared to 19.9).^16^ Tennessean women experience the 12th highest BC mortality-to-incidence ratio in the U.S.^19^ Stage at diagnosis is the first intervenable time point to improve mortality outcomes in BC patients.

Given the high BC mortality rate, documented obstacles to preventive health services in Appalachia, and benefits of early diagnosis, it is essential to understand the geographic and sociodemographic factors associated with invasive BC among Tennessean women. Despite this need and Tennessee's unique Appalachian geography, limited literature has investigated BC disparities within the state.

Our aim was to assess whether sociodemographic factors, health insurance status, and county of residence (Appalachian vs. non-Appalachian) were associated with invasive BC using the Tennessee Cancer Registry (TCR). We further evaluated these associations in subpopulations of women from Appalachian and non-Appalachian counties. Findings will provide important information about how interventions should be tailored to reduce BC disparities among women in Tennessee.

## Materials and Methods

### Data source and study population

Between 2005 and 2015 52,187 BC cases were recorded in the TCR. The TCR is a population-based, central cancer registry serving the citizens of Tennessee and was established by Tennessee law to collect and monitor cancer incidence.^20^ The study population includes all Tennessee women residents aged ≥18 years who were diagnosed with histologically confirmed BC as the primary site of diagnosis as coded by the International Classification of Diseases for Oncology, Third Edition (ICD-O-3), and reported to the TCR between January 1, 2005 and December 31, 2015.

Regression analyses were performed on 42,966 invasive BC cases, and 9221 non-invasive cases. Data used for this analysis are restricted but available by request to the Tennessee Department of Health (TDH), TCR.^21^ All analytical files are available by reasonable request. The TDH Institutional Review Board approved the research protocol.

### Dependent variable

Using the North American Association of Central Cancer Registries (NAACCR) Standards for Cancer Registries Volume II Data Standards and Data Distortionary, the outcome variable was invasive BC as defined by ICD-O-3, histology code and behavior code 3. The ICD-O-3 behavior code for *in situ* in the NAACCR dictionary is coded as “2” and invasive as “3.”^22^

### Independent variables

Individual-level sociodemographic variables obtained from TCR included race, age at diagnosis, marital status, type of health insurance, and county of residence. Age was categorized as <45; 45–54; 55–64; and ≥65 years, and race as Black and White. Marital status was categorized as single/never married; married/common-law; divorced/separated; and widowed. Type of insurance was categorized as public (Medicaid, Medicare, Indian Health Service, Veterans' Affairs); private (fee for service, Health Maintenance Organization, Managed Care, and Preferred Provider Organization); and self-pay or uninsured. County of residence was categorized as Appalachian or non-Appalachian as illustrated by Figure 1.

### Statistical analysis

We first summarized each variable as follows: we obtained the distribution of the continuous age variable by mean, standard deviation (SD), and range (lower and upper age at diagnosis). The distribution of the categorical factors was summarized using frequencies and percentages.

Next, we performed multivariable logistic regression on our sample (*N* = 52,187) to examine the associations between invasive BC diagnosis and race, age at diagnosis, marital status, county of residence, and health insurance coverage. We employed backward stepwise regression to carefully select the significant variables/covariates associated with the response. Finally, we stratified our sample by county of residence and performed two stratified multivariable logistic regressions with the aforementioned independent variables on subpopulations of women residing in (1) Appalachian (*n* = 24,501) and (2) non-Appalachian (*n* = 27,686) counties.

The results of the logistic regressions are reported based on the independent variable coefficient, adjusted odds ratio (OR), 95% confidence interval (CI), and statistical significance. All analyses were conducted using SAS statistical software, version 9.4 (Cary, NC).

## Results

### Sample characteristics

Sociodemographic characteristics for the whole sample are displayed in Table 1. Age of patients ranged from 20 to 105 years with a mean age of 61.18 years, and an SD of 13.02. Most women in the sample (85.45%, *n* = 44,596) were White, married (58.50%, *n* = 30,528), and diagnosed with invasive BC (82.33%, *n* = 42,966). An almost equal number of the women in the sample had public health insurance (50.46%, *n* = 26,332) or private health insurance coverage (48.37%, *n* = 25,242) while less than 2% (*n* = 613) reported no health insurance. See Table 1 for more information. Of the 42,966 invasive cases, more than half (52.96%, *n* = 22,754) were recorded in non-Appalachian counties whereas 47.04% (*n* = 20,212) were in Appalachian counties.

**Table 1.** Characteristics of Tennessee Women Aged ≥18 Years Diagnosed with Breast Cancer, 2005–2015, ***N*** **= 52,187**

	Appalachia		Non-Appalachia		Overall	
	***n***	%	***n***	%	***n***	%
	24,501	46.95	27,686	53.05	52,187	100.00
Age group (years)						
<45	2243	9.15	3254	11.75	5497	10.53
45–54	4684	19.12	6410	23.15	11,094	21.26
55–64	6661	27.19	7555	27.29	14,216	27.24
≥65	10,913	44.54	10,467	37.81	21,380	40.97
Race						
White	23,252	94.90	21,344	77.09	44,596	85.45
Black	1249	5.10	6342	22.91	7591	14.55
Marital status						
Single/never married	2664	10.87	4165	15.04	6829	13.09
Married/common law	14,742	60.17	15,786	57.02	30,528	58.50
Divorced/separated	2820	11.51	3326	12.01	6146	11.78
Widowed	4275	17.45	4409	15.93	8684	16.64
Health insurance type						
Self-pay/uninsured	262	1.07	351	1.27	613	1.17
Public	12,759	52.08	12,483	45.09	25,242	48.37
Private	11,480	46.86	14,852	53.64	26,176	50.46
Breast cancer stage						
Invasive	20,212	82.49	22,754	82.19	42,966	82.33
Non-invasive	4289	17.51	4932	17.81	9221	17.67

	**Mean**	**SD**	**Mean**	**SD**	**Mean**	**SD**
Age at diagnosis (range: 20, 105)	62	12.84	60	13.11	61	13.02

Public Insurance (Indian Health Service, Medicaid, Medicare, Veterans' Affairs). Private Insurance (Fee for services, HMO, Managed Care, PPO).

HMO, Health Maintenance Organization; PPO, Preferred Provider Organization; SD, standard deviation.

### Multivariable logistic regression: combined Appalachian and non-Appalachian population

The results of the multivariable logistic regression revealed age at diagnosis, marital status, and health insurance coverage are associated with invasive BC among Tennessean women with BC (Table 2). Patients aged <45 (OR = 0.74; 95% CI = 0.67–0.81; *p* < 0.0001) and 55–64 years (OR = 0.91; 95% CI = 0.84–0.97; *p* = 0.006) had decreased odds of having invasive BC compared to women ≥65 years. A positive association with invasive stage diagnosis was seen among married women (OR = 1.12; 95% CI = 1.04–1.21; *p* = 0.002) and women with both public (OR = 1.81; 95% CI = 1.41–2.33; *p* < 0.0001) and private health insurance coverage (OR = 1.36; 95% CI = 1.06–1.76; *p* = 0.018).

**Table 2.** Multivariable Logistic Regression Analysis of Sociodemographic and Health Insurance Factors Associated with Invasive Breast Cancer in Appalachian and Non-Appalachian Women Aged ≥18 with Breast Cancer, ***N*** **= 52,187**

Variable description	Coefficient	SE	***p***	OR (95% CI)
Constant	−1.96	0.134	<0.0001	0.141
Age group (years)				
<45	**−0.300**	**0.048**	**<0.0001**	**0.74 (0.67–0.81)**
45–54	−0.044	0.039	0.257	0.96 (0.89–1.03)
55–64	**−0.099**	**0.035**	**0.006**	**0.91 (0.84–0.97)**
≥65	—	—	—	—
Race				
White [Ref.]	—	—	—	—
Black	0.019	0.035	0.588	1.02 (0.95–1.09)
Marital status				
Single/never married [Ref.]	—	—	—	—
Married/common-law	**0.115**	**0.037**	**0.002**	**1.12 (1.04–1.21)**
Divorced/separated	**−0.106**	**0.048**	**0.032**	**0.92 (0.82–0.99)**
Widow	**−0.151**	**0.047**	**0.001**	**0.86 (0.78–0.94)**
County of residence				
Appalachian	−0.010	0.024	0.662	0.99 (0.9–1.04)
Non-Appalachian [Ref.]	—	—	—	—
Health insurance type				
Self-pay/uninsured [Ref.]	—	—	—	—
Public	**0.594**	**0.128**	**<0.0001**	**1.81 (1.41–2.33)**
Private	**0.308**	**0.130**	**0.018**	**1.36 (1.06–1.76)**

Public Insurance (Indian Health Service, Medicaid, Medicare, Veterans' Affairs). Private Insurance (fee for services, HMO, Managed Care, PPO).

Bold = Statistically significant *p* < 0.05.

CI, confidence interval; OR, odds ratio; SE, standard error.

### Multivariable logistic regression: Appalachian subpopulation

The results of the subpopulation analyses are displayed in Table 3. For women residing in Appalachian counties, we found that compared to women aged ≥65 years, women under age 45 had 27% decreased odds (95% CI = 0.63–0.84; *p* = 0.0001) of presenting with invasive BC. Women aged 45–54 and 54–64 years did not have significantly different odds of invasive BC than women 65 years or older. Also, married/common-law women were more likely than single/never married women to present with invasive stage disease (OR = 1.13; 95% CI = 1.01–1.26; *p* = 0.036). However, there was no difference with invasive BC presentation among divorced/separated (95% CI = 0.81–1.08; *p* = 0.369) and widowed women (95% CI = 0.76–1.00; *p* = 0.053) in comparison to single/never-married women.

**Table 3.** Stratified Multivariable Logistic Regression Analyses for Sociodemographic and Health Insurance Factors Associated with Invasive Breast Cancer in Subpopulations of Appalachian and Non-Appalachian Women Aged ≥18 with Breast Cancer in Tennessee

Factors	Appalachian counties (***N*** = 24,501)				Non-Appalachian counties (***N*** = 27,686)			
	Coefficient	SE	***p***	OR (95% CI)	Coefficient	SE	***p***	OR (95%CI)
Constant	−1.761	0.189	<0.0001	0.172	−2.143	0.192	<0.0001	0.117
Age group (years)								
<45	**−0.316**	**0.072**	**0.0001**	**0.73 (0.63–0.84)**	**−0.302**	**0.065**	**<0.0001**	**0.74 (0.65–0.84)**
45–54	−0.005	0.056	0.936	1.00 (0.85–1.11)	−0.084	0.055	0.127	0.92 (0.83–1.02)
55–64	−0.067	0.051	0.184	0.94 (0.91–1.03)	**−0.133**	**0.051**	**0.009**	**0.88 (0.79–0.97)**
≥65 [Ref.]	—	—	—	—	—	—	—	—
Race								
White [Ref.]	—	—	—	—	—	—	—	—
Black	0.058	0.077	0.435	1.06 (0.91–1.23)	0.013	0.039	0.741	1.01 (0.94–1.10)
Marital status								
Single/never married [Ref.]	—-	—	—	—	—	—	—	—
Married/common-law	**0.120**	**0.057**	**0.036**	**1.13 (1.01–1.26)**	**0.110**	**0.048**	**0.022**	**1.12 (1.02–1.23)**
Divorced/separated	−0.066	0.074	0.369	0.94 (0.81–1.08)	**−0.136**	**0.064**	**0.033**	**0.88 (0.77–0.99)**
Widow	−0.137	0.071	0.053	0.88 (0.76–1.00)	**−0.167**	**0.064**	**0.009**	**0.85 (0.75–0.96)**
Health insurance type								
Self-pay/uninsured [Ref.]	—	—	—	—	—	—	—	—
Public	**0.354**	**0.179**	**0.049**	**1.42 (1.00–2.03)**	**0.812**	**0.185**	**<0.0001**	**2.25 (1.57–3.24)**
Private	0.072	0.182	0.693	1.07 (0.75–1.53)	**0.519**	**0.187**	**0.006**	**1.68 (1.16–2.24)**

Public Insurance (Indian Health Service, Medicaid, Medicare, Veterans' Affairs). Private Insurance (fee for services, HMO, Managed Care, PPO).

Bold = Statistically significant *p* = < 0.05.

A slightly positive association was seen with public health insurance coverage (OR = 1.42; 95% CI = 1.00–2.03; *p* = 0.049) when compared to self-pay/uninsured women. No statistically significant association was found between private health insurance coverage and invasive BC diagnosis nor was there an association found with race.

### Multivariable logistic regression: non-Appalachian subpopulation

Nearly all sociodemographic factors, except race, were shown to be significantly associated with invasive BC diagnosis among women in non-Appalachian Tennessee (Table 3). Compared to women aged ≥65 years, women under age 45 and those aged 55–64 had 26% (95% CI = 0.65–0.84; *p* < 0.0001) and 12% (95% CI = 0.79–0.97; *p* = 0.009) decreased odds, respectively, of presenting with invasive BC. No statistically significant difference was observed for women aged 45–54 years compared to women aged ≥65 regarding invasive BC diagnosis in non-Appalachian counties.

Marital status was also significantly associated with invasive BC. Married/common-law women were more likely than single/never married women to present with invasive stage disease (OR = 1.12; 95% CI = 1.02–1.23; *p* = 0.022). Rather, divorced/separated and widowed women had 13% (95% CI = 0.77–0.99; *p* = 0.033) and 15% (95% CI = 0.75–0.96; *p* = 0.009) reduced odds, respectively, of presenting with invasive BC in comparison to single/never-married women.

A positive association with invasive stage diagnosis was seen with public health insurance coverage (OR = 2.25; 95% CI = 1.57–3.24; *p* < 0.0001) and private health insurance (OR = 1.68; 95% CI = 1.16–2.24; *p* = 0.006) when compared to self-pay/uninsured women. No statistically significant association was found between race and invasive BC diagnosis.

## Discussion

Our analysis found that younger and divorced/separated and widowed women were less likely to be diagnosed with invasive BC compared to older and single/never married women in Tennessee. Additionally, we found married women and women on public or private insurance to have greater odds of invasive BC than single/never married women and uninsured/self-pay women. We found no association between Appalachian county residence and invasive BC diagnosis. These findings contribute to the sparse literature on cancer disparities in Tennessee.

Previous studies^15,23–26^ report high cancer rates and health disparities in rural America, particularly in the Appalachian region. Despite this, we found no association between Appalachian county residence and invasive BC diagnosis in Tennessee. This finding aligns with a recently published study that investigated geographic differences in late stage BC diagnosis in Tennessee.^27^ However, the study also found that of the 9 Tennessee counties with the highest incidence rates, six fall within Appalachia.^27^

Yao et al.^11^ also found regional BC disparities using Surveillance, Epidemiology, and End Results (SEER) Program data. The study found that the proportion of female BC patients receiving early diagnoses in rural and urban Appalachia was lower than in corresponding non-Appalachian regions.^11^

Our null findings were contrary to our hypothesis that Appalachian Tennessee county residence would be associated with invasive BC diagnosis due to barriers to preventive health services, high rates of obesity and smoking, and high poverty in Appalachia. In an analysis of three Appalachian states (Pennsylvania, Ohio, and Kentucky), Anderson et al.^26^ found that counties with the highest economic deprivation also reported the highest late-stage diagnosis. However, another SEER study found that disparities in cancer incidence between Appalachian and non-Appalachian regions has narrowed, except for oral cavity and pharynx, larynx, lung and bronchus, and thyroid cancers.^14^

Our study found younger age to be negatively associated with invasive BC diagnosis. This finding is consistent with increasing age as a documented BC risk factor.^16^

We additionally found no significant difference in invasive BC diagnosis between Black and White women in Tennessee. A finding that is consistent with another recently published study.^27^ Despite this result, racial disparities in BC mortality in the U.S. persist at striking levels. While BC incidence rates among non-Hispanic White and Black women are similar, Black women have a 41% higher mortality rate.^16^

A study that investigated BC morality by race in the 25 largest U.S. cities reported that in Memphis, a non-Appalachian city in Tennessee, Black women had a mortality rate 2.09 times that of White women, the most extreme disparity of any city included in the study.^28^ While we did not measure mortality, later stage diagnosis is associated with lower survival rates.^16^ Notably, between 2008 and 2012 BC incidence rates were markedly higher among Black women compared to White women in the southern U.S.^29^

Studies^30–39^ have demonstrated lower BC risk and mortality for married women compared to single/unmarried women. Factors attributed to this finding include the financial stability, engagement in physical activity, and healthy lifestyle behaviors (better nutrition, family support, adherence to recommended screening, and treatment) observed among married women.^30–34^

Nevertheless, our study showed that married Tennessean women had slightly higher odds of invasive BC diagnosis than single women. Among possible explanations for our finding is that married women in Tennessee prioritize family responsibilities and financial concerns over their own health and in turn delay seeking care, including preventive health services thus contributing to a higher likelihood of invasive BC diagnosis.

Existing literature has primarily studied overall BC risk in relation to tumor types, neighborhood socioeconomic status, mortality, and survival using SEER registries, our study involved late-stage diagnosis from a region that is not part of the SEER database. This offers a possible reason for our unique findings in relation to marital status. For instance, Hsu et al.^31^ used SEER 18 cancer registries that include 12 states including Appalachian states Georgia and Kentucky. Gomez et al.^30^ and Martínez et al.^33^ evaluated the California Cancer Registry, which is also included in the SEER registries. More research is needed to understand the relationship between marriage and invasive BC.

Equitable access to quality health care is one of the major necessities to promoting health and well-being in minority and underserved populations, especially in poor and rural areas.^40^ Evidence indicates that inequalities in health care access are primarily due to high numbers of not insured or underinsured low-income individuals.^31,41–45^ For instance, several past studies^31,42–45^ have found that uninsured patients and patients on Medicaid were more likely to receive a later stage BC diagnosis than women with private insurance.

Notably, our analysis revealed that women with both public and private health insurance are significantly more likely to be diagnosed with invasive disease when compared to self-pay/uninsured women. This result may be partially explained by the findings of a systematic review by Agarwal et al.^46^ that reported that although high-deductible health plans are associated with lower premiums, the high out-of-pocket payments were related to a reduction in office-visits, preventive services, and medication adherence among patients. Al Rowas et al.^47^ also found that private health insurance is associated with a delay in seeking care and treatment due to high cost-related co-pays and deductibles compared to publicly insured individuals (15.6% vs. 8.1% respectively).

Our findings serve to provide important information as to the public health needs of residents of Tennessee and the Appalachian region broadly. Regular mammography can lead to earlier stage at diagnosis and improved outcomes for BC patients. As such, interventions should promote mammography utilization among women in Tennessee.

General mistrust and fatalism toward the health care system is pervasive in Appalachia and could lead to underutilization of preventive services.^3^ Intervention programs should appeal to the strong regional faith in Appalachia by partnering with churches in an effort to build trust.^3,5^ This is a particularly relevant approach to reaching older and married women who we found to be more vulnerable to later stage diagnosis. Trust building among the insured population may also be key to improving diagnostic outcomes in this group. More research is needed on the specific barriers married and insured women may face to receiving timely screening services.

Our study adds to current BC literature by assessing the relationship between sociodemographic characteristics, health insurance coverage, and Appalachian residence designation in a geographically unique state not currently covered in the SEER program. However, there are some limitations to this study. First, the results are not generalizable to the entire U.S. population because the TCR only collects cancer data on Tennessee residents and individuals that receive treatment in Tennessee. The study is additionally limited by the retrospective administrative variables available. For instance, important confounding variables (*e.g.*, education level, income status) and changes in sociodemographic variables such as marital status, place of residence, and health insurance could not be controlled for.

The inability to control for the aforementioned possible confounders along with family history and behavioral/lifestyle factors may help to explain our finding that insured and married women have higher odds of being diagnosed with invasive stage disease. Additionally, due to confidentiality concerns we were unable to differentiate between individuals on Medicare and Medicaid. Notwithstanding these limitations, findings are important because they provide a better understanding of the category of women most likely to be diagnosed with invasive BC in Tennessee.

## Conclusions

BC survival rates are high when diagnosis and treatment happen early. This study is among the first to investigate BC diagnosis by Appalachian county designation in Tennessee. Age at diagnosis, marital status, and health insurance coverage were associated with invasive stage disease. Further research is necessary to determine why public and private insurance coverage, besides being married, was associated with worse diagnosis outcomes in Tennessee. Additionally, further research on racial disparities in BC diagnosis is needed in this region.

Early detection and screening services could improve rates of early diagnosis. However, the sociodemographic and geographic characteristics of the Appalachian region make residents particularly vulnerable to falling through existing preventive safety nets. Interventions should target all women in Tennessee and focus on instilling trust in health care providers and reducing fatalism.

## Data Sharing Statement

Data used for this analysis are restricted but available by request to the TDH, TCR, https://www.tn.gov/education/data/data-downloads/request-data.html.

## Abbreviations Used

BC: breast cancer
CI: confidence interval
HMO: Health Maintenance Organization
ICD-O-3: International Classification of Diseases for Oncology, Third Edition
IRB: Institutional Review Board
NAACCR: North American Association of Central Cancer Registries
NIH: National Institutes of Health
OR: odds ratio
PPO: Preferred Provider Organization
SD: standard deviation
SE: standard error
SEER: Surveillance, Epidemiology, and End Results
TCR: Tennessee Cancer Registry
TDH: Tennessee Department of Health
